# Heart Rate and Blood Pressure Centile Curves and Distributions by Age of Hospitalized Critically Ill Children

**DOI:** 10.3389/fped.2017.00052

**Published:** 2017-03-17

**Authors:** Danny Eytan, Andrew J. Goodwin, Robert Greer, Anne-Marie Guerguerian, Peter C. Laussen

**Affiliations:** ^1^Department of Critical Care Medicine, The Hospital for Sick Children, University of Toronto, Toronto, ON, Canada; ^2^Department of Pediatric Critical Care, Rambam Medical Center, Haifa, Israel; ^3^Neuroscience and Mental Health Program, Research Institute, Toronto, ON, Canada; ^4^Department of Anaesthesia, University of Toronto, Toronto, ON, Canada

**Keywords:** pediatrics, critical care, heart rate, blood pressure, percentiles, big data

## Abstract

Heart rate (HR) and blood pressure (BP) form the basis for monitoring the physiological state of patients. Although norms have been published for healthy and hospitalized children, little is known about their distributions in critically ill children. The objective of this study was to report the distributions of these basic physiological variables in hospitalized critically ill children. Continuous data from bedside monitors were collected and stored at 5-s intervals from 3,677 subjects aged 0–18 years admitted over a period of 30 months to the pediatric and cardiac intensive care units at a large quaternary children’s hospital. Approximately 1.13 billion values served to estimate age-specific distributions for these two basic physiological variables: HR and intra-arterial BP. Centile curves were derived from the sample distributions and compared to common reference ranges. Properties such as kurtosis and skewness of these distributions are described. In comparison to previously published reference ranges, we show that children in these settings exhibit markedly higher HRs than their healthy counterparts or children hospitalized on in-patient wards. We also compared commonly used published estimates of hypotension in children (e.g., the PALS guidelines) to the values we derived from critically ill children. This is a first study reporting the distributions of basic physiological variables in children in the pediatric intensive care settings, and the percentiles derived may serve as useful references for bedside clinicians and clinical trials.

## Introduction

Variables such as heart rate (HR), respiratory rate, and blood pressure (BP) form the basis for evaluating and monitoring the physiological state of patients. These variables may be recorded continuously and serve for detecting deterioration, modifying interventions, and assessing response to treatments in sick children. Although norms have been determined in healthy and hospitalized children (1–3), for acutely-ill children hospitalized in the critical care unit, there is a severe knowledge gap with no published data to the best of our knowledge.

Currently, critical care clinicians set target ranges manually according to the assessed clinical state or resort to using published norms derived from the studies of healthy children, at rest, in a sitting position or ambulating, using devices and measurement methods intended to be used in out-patient settings. Norms generated from estimates obtained in healthy subjects were most often not cross-validated in the decubitus position, across the broad physiologic and disease conditions experienced during childhood critical illness or for variables recorded using invasive monitoring devices. So, it is hard to extrapolate from the healthy condition to the expected values in the intensive care setting. Importantly, for BP, the purpose of the majority of the studies in healthy children was to develop normative data to operationalize the diagnosis of hypertension and not to generate thresholds to define hypotension.

Having normative data generated in a representative population is instrumental to producing valid thresholds; these could be applied to define deterioration such as hypotension, to assess response to treatment with medications and medical devices applied to treat shock, or to facilitate endpoints in clinical trials conducted in children (e.g., to set hypotension thresholds for drug trials). The gap in appropriate normative values may be associated with the undertreatment or overtreatment of children during critical illness (4–7). To start to fill this gap, we set out to generate centile curves for HR and BP in critically ill children as a function of age.

We hypothesized that pediatric published norms for physiological variables derived from measurements taken from healthy and hospitalized children are different than the distributions of these variables during critical illness and that these variables do not follow a Gaussian distribution. Even though for any given patient the HR and BP are obviously influenced by many factors (e.g., illness severity, underlying diagnoses, ventilator, and pharmacologic support), as this is a first study aimed at beginning to fill the above-described knowledge gap for critically ill children, we chose to focus on the distributions conditional only to age at the time of recording and to compare these to commonly used published references such as the PALS guidelines.

We used an electronic signal repository containing all recorded physiological variables from the clinical bedside monitoring units for all patients admitted to The Hospital for Sick Children’s Critical Care Department since April 2013. These data allowed generation of detailed distributions of physiological variables and to compare the derived reference ranges with existing published ranges.

## Materials and Methods

This is an observational descriptive study consisting of a retrospective retrieval of prospectively recorded physiologic data of patients younger than 18 years hospitalized at The Hospital for Sick Children between April 1, 2013, 00:00:00 and September 30, 2015, 23:59:59 in either the pediatric intensive care unit (PICU) or the cardiac critical care unit. Data from all consecutive patients that conformed to the criteria detailed below were included for analysis. Patients admitted during this time were all included unless they had less than 10,000 values on record (minimal length of stay of ~14 h), were older than 18 years, or whose files were corrupted and unreliable. This resulted in an exclusion of ~10% of the patients, mainly due to short length of stay. Physiological variables recorded by the bedside monitors (Intellivue MP70, Philips, Amsterdam, Netherlands, software version J.10.50) have been stored at 5-s intervals using T3 risk monitoring (Etiometry, Boston, MA, USA) on a secure institutional server, and this large database served as the basis for the current report. In this study, we focus on HR and invasively recorded arterial BP as these are basic physiological variables used for clinical assessment, predictions, and alarm settings, and they are less prone to be influenced by artifacts compared to variables such as central venous pressure, which may be skewed due to additional infusions or respiratory rate as many of these patients were ventilated throughout parts of their hospitalization. These data were combined with additional information from each patient’s electronic health record such as date of birth. Of note, the HR data were derived from the monitor’s continuous stream of HR as calculated from the ECG leads, whereas the BP data were calculated and reported by the monitor using invasive intraarterial BP tracings only.

### Data Quality

Several measures were undertaken to ensure data quality and integrity. For each patient, HR or BP data were included for analysis only if that patient had at least 10,000 values on record (spanning a course of ~14 h of recording). Similarly to what was reported by Bonafide et al. (2), for either HR or BP, data points that were deemed to be implausible were discarded (≤0 or ≥300). For HR data, we also discarded time periods in which the patient was electrically paced. These time periods were identified using an artificial neural network filter implemented in MATLAB (The MathWorks, Natick, MA, USA) with a single hidden layer and an accuracy of 98.2%. The network was trained and tested on a data set derived from a sample set of 200 patients. Threshold for filtering was set such that we correctly included 98% of all non-paced samples and correctly excluded 92% of all paced samples.

### Distribution Generation

For each physiological variable and age group, we generated the data-derived probability distribution in the following way.

To allow detailed examination of the trends in vital signs in the first weeks of life on the one hand and to allow comparison with previous studies examining physiological variables percentiles for healthy and hospitalized children (1, 2), we chose the following 43 target age groups: each of the first 30 days of life, 0–3 months, 3–6 months, 6–9 months, 9–12 months, 12–18 months, 18–24 months, 2–3 years, 3–4 years, 4–6 years, 6–8 years, 8–12 years, 12–15 years, and 15–18 years. The age groups spanning either months or years were non-overlapping: for example, the age group 18–24 months contains children who were up to (and including) exactly 2 years of age at the time of recording, and the following age group of 2–3 years contains all children who were a day older than 2 years and up to 3 years of age at the time of recording.

Observations from each patient were partitioned into patient age group subsets representing one or more of the 43 age groups depending on the age of the patient at the time each individual observation was recorded. Some patients may have contributed information to more than one age group. Repeated admissions were included, with only the patient age considered when including an observation in a given subset.

**Figure 1:**
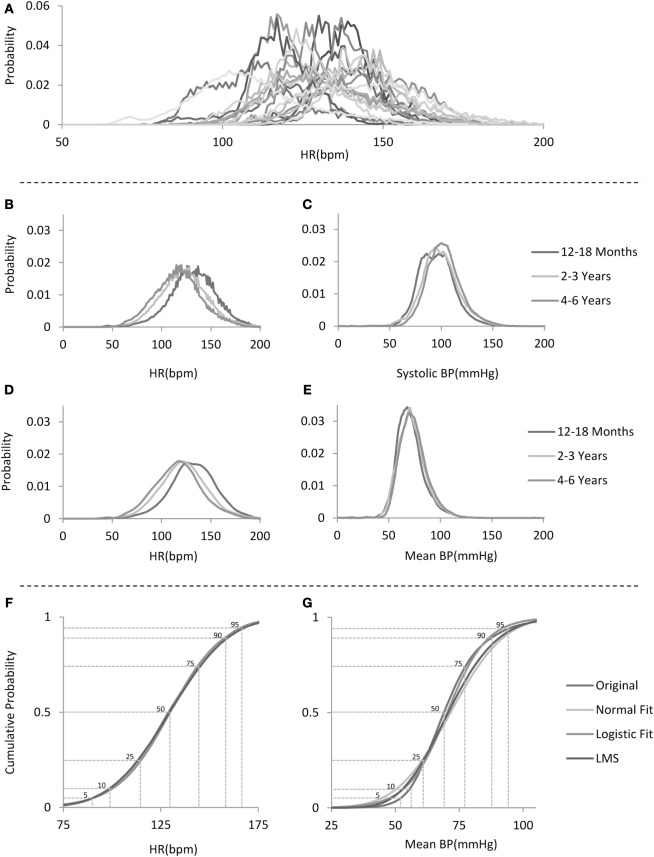
**Analysis flow**. **(A)** Example distributions of heart rate (HR) for 20 individual patients in the 12–18 months age group. **(B)** Sample non-smoothed distributions for HR for children in the three age groups depicted on the right. **(C)** Sample systolic blood pressure (BP) distributions for children in the same age groups as in **(B)**. **(D)** Sample smoothed distributions for HR for children in the same three age groups as in **(B)**. **(E)** Sample mean BP distributions for the same age groups as in **(B)**. Note the narrower and skewed distribution shape. **(F,G)** Sample cumulative distribution functions for HR and mean BP for the 12–18 months age group with comparison to several fitted standard distributions with percentile lines overlaid.

Any patient age group subset that contained less than 10,000 observations was excluded from analysis. To avoid sampling bias due to different lengths of stay in the ICUs (and therefore different number of contributing values), which would lead to population-level distributions heavily weighted to represent those patients with longer stays, for each patient, a normalized distribution was generated to produce a single patient age group distribution (see examples of individual patient age group distributions of HRs from the 12– 18 months age group in Figure 1A), and these in turn were combined within the age groups and renormalized to produce the age group-specific distributions. Thus, for every age group, each patient contributed the same weight in generating the distribution, regardless of the number of data points recorded from that patient. Figure 1B depicts the age group distributions of HRs for three example age groups, whereas Figures 1C,E depict the respective systolic and mean BP distributions for the same example age groups.

Of note, as can be seen in Figure 1B (sample HR distributions for three age groups), there seems to be a subtle digit preference for certain values as manifested by the “sawtooth” pattern of the data-derived probability distribution. We speculate that this is probably due to the algorithm used by the bedside monitor to calculate the HR per minute as it was observed for the same values in all patients and age groups. Figure 1D shows HR distributions that have been smoothed by using a locally weighted scatter plot method (8). We report for each derived unsmoothed distribution, its skewness and kurtosis.

**Figure 2:**
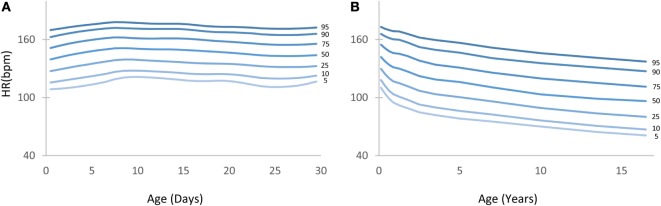
**Heart rate (HR) centile curves as a function of age**. **(A)** HR centile curves for children aged 0–30 days depicting the 5th to 95th percentiles. **(B)** HR centile curves for children aged 0–18 years.

**Figure 3:**
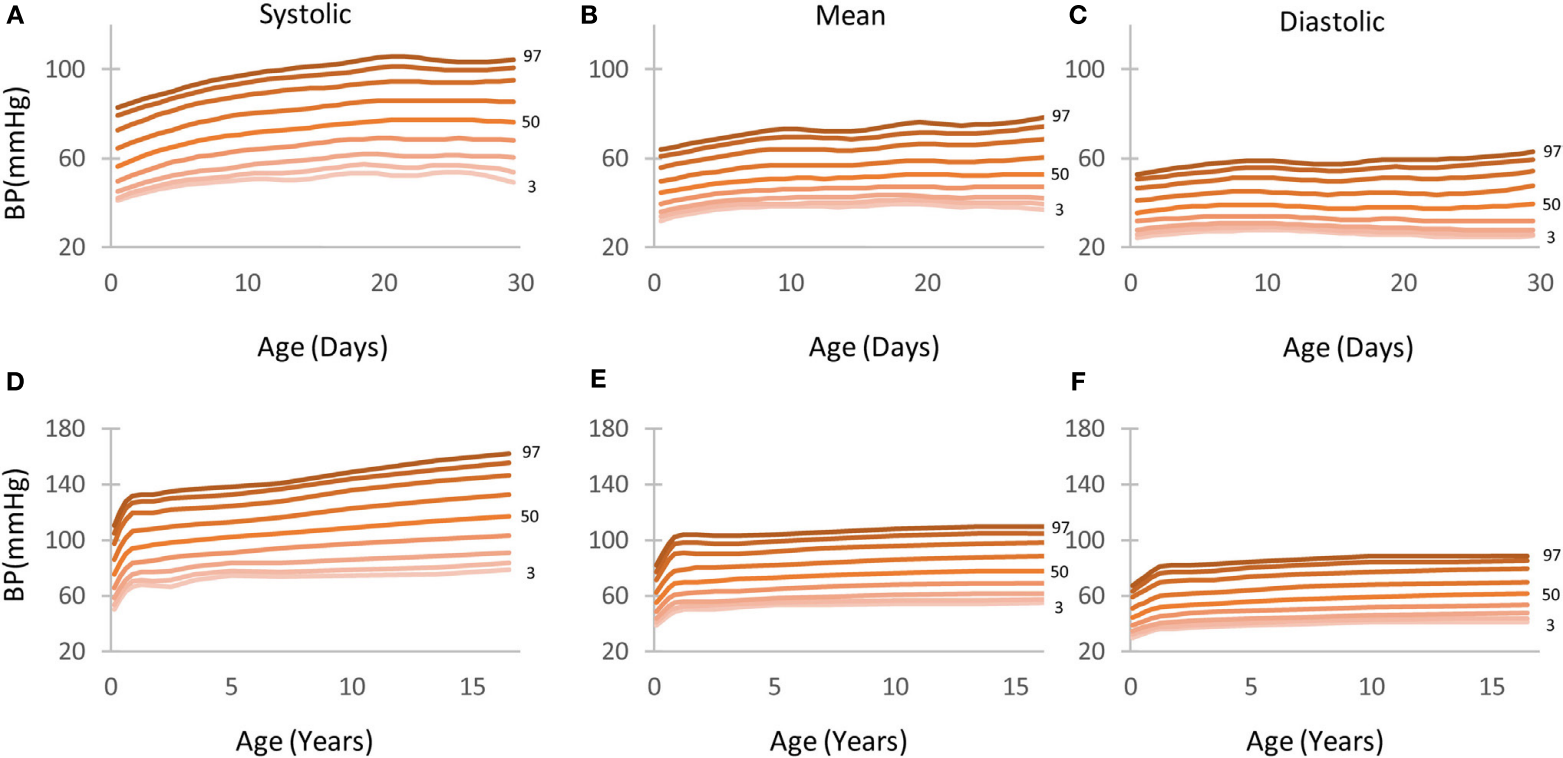
**Arterial blood pressure (BP; systolic, mean, and diastolic) centile curves by age depicting the 5th to 95th percentiles**. **(A–C)** Children aged 0–30 days. **(D–F)** children aged 0–18 years.

For each age group distribution, we directly calculated the cumulative distribution function and compared these to fitted standard distributions (Figures 1F,G) (1, 2, 9–13). Gaussian and Logistic estimates were derived using regression based on maximum likelihood estimates. Additional percentile curves were derived based on probability distribution estimates either by the non-parametric method of quantile regression (14, 15) or the parametric Lamda-mu-sigma (LMS) implemented in MATLAB (see Figures 1F,G for sample cumulative distributions derived from the data and their respective estimates using some of the above-mentioned probability distributions). For each age group, from the cumulative distribution function, we derived the percentiles (1st to 99th) and used these to generate centile curves smoothed over the age groups using a locally weighted scatter plot method (shown in Figures 2 and 3; Tables S1–S8 in Supplementary Material).

This study was approved by The Hospital for Sick Children’s Research Ethics Board in Toronto, ON, Canada. As this was a retrospective analysis of anonymized data from thousands of patients, the requirement for informed consent was waived by the institution’s research ethics board.

## Results

A total of 1,126,549,078 data points were extracted from 3,677 patient episodes for care. Table 1 shows the average number of patients and observations for children in either one of the first 30 days of life age groups or the 13 age groups comprising 0–18 years of age that were used to generate the centile curves for either HR or BP. Importantly, BP measurements were derived only from the intraarterial recordings. The distributions for each age subgroup comprise a combination of 212 ± 109 and 134 ± 86 individual patient age group distributions for HR and BP, respectively; for patients in the 1–30 days of life age groups (total of 30 age groups), an average 13,375 and 14,096 data points were used for each individual patient’s distribution generation for HR and BP, respectively, whereas for patients in the 0 months to 18 years of life age groups (total of 13 age groups), an average 94,805 and 64,321 data points were used for each individual patient’s distribution generation for HR and BP, respectively.

**Table 1:** **Study population statistics**.

	Age groups	Average number of patients per group	Average number of observations per group	% male
Heart rate	0–30 days	166	2,224,349	56
	0–18 years	317	30,024,184	54
Blood pressure	1–30 days	104	1,461,373	59
	0–18 years	214	13,794,392	53

### HR Percentiles

Figure 2 depicts the HR centile curves for each age group with Figures 2A,B showing the 5th to 95th curves for the first 30 days and the 0–18 years divisions, respectively.

Table S1 in Supplementary Material shows in detail the 1st to 99th HR percentiles for children aged 0–18 years. A detailed table showing percentiles for children aged 0–30 days can be found in the Table S2 in Supplementary Material. The 5th and 95th percentile columns are highlighted in gray for ease of reference.

### BP Percentiles

Figure 3 depicts the BP centile curves (5th to 95th) as calculated for the first month (Figures 3A–C) and the 0–18 years division (Figures 3D–F).

Tables S3–S5 in Supplementary Material show in detail the 1st to 99th BP (systolic, mean, and diastolic) percentiles for children aged 0–18 years. Detailed tables showing percentiles for children aged 0–30 days can be found in the Tables S6–S8 in Supplementary Material. The 5th and 95th percentile columns are highlighted in gray for ease of reference.

### Kurtosis and Skewness of the Distributions

An underlying assumption in most studies aimed at generating normative values for physiological variables is that these follow an approximately Gaussian distribution. The large number of data points available for analysis in this study allowed us to test this assumption directly. For example, examining the cumulative distributions of the HR and BP, as can be seen in the samples presented in Figures 1F,G, illustrates their properties: although the HR cumulative distribution (marked as Original) is closely matched by any of the fitted standard statistical models, this is not the case for the mean arterial BP. The mean arterial BP distributions reveal that they have a sharper peak or a positive kurtosis (leptokurtic) and are positively skewed, as can be seen for example in Figure 1E. Table 2 provides statistics on the excess kurtosis and skewness of all distributions: both the HR and systolic BP distributions for all ages are relatively symmetric and mesokurtic, in contrast to the mean and diastolic BP distributions that exhibit a sharper shape (leptokurtic) and are more positively skewed.

**Table 2:** **Kurtosis and skewness descriptive statistics**.

	Excess kurtosis			Skewness		
	Mean	SD	Interquartile range	Mean	SD	Interquartile range
Heart rate (HR) 0–30 days	1.06	0.82	0.57–1.2	−0.21	0.23	−0.4 to −0.12
HR 0–18 years	0.31	0.29	0.09–0.64	0.14	0.18	0.06–0.28
Blood pressure (BP) systolic 30 days	1.35	1.03	0.79–1.48	0.41	0.24	0.26–0.53
BP systolic 0–18 years	0.91	0.44	0.60–1.06	0.38	0.19	0.28–0.44
BP mean 30 days	62.50	15.55	55.37–71.33	4.95	0.96	4.62–5.61
BP mean 0–18 years	22.50	7.21	17.11–24.91	2.46	0.62	1.96–2.66
BP diastolic 30 days	5.53	4.03	3.06–6.02	1.02	0.22	0.91–1.12
BP diastolic 0–18 years	4.26	1.28	3.63–4.69	1.04	0.14	0.97–1.11

### Comparison to Published Reference Ranges

**Figure 4:**
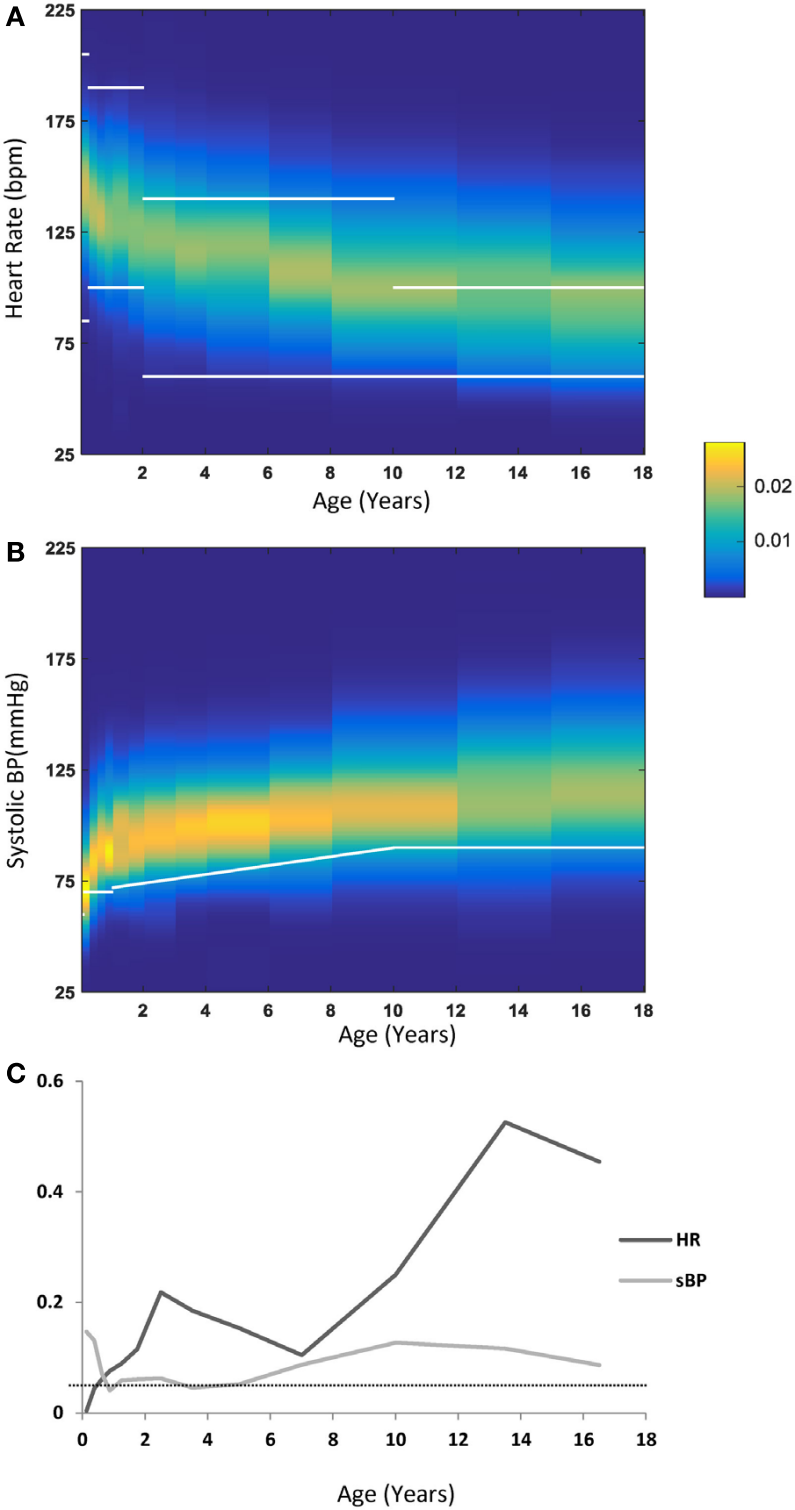
**(A)** Heart rate (HR) distribution by age—each vertical band represents one age group, with the probability of each HR value coded by the color map detailed in the legend. Overlaid in white lines are the cutoff ranges as published in the PALS guidelines. **(B)** Systolic blood pressure (sBP) distributions by age with a similar representation scheme as in **(A)**. Overlaid in a white line are the lower cutoff values as published in the PALS guidelines. **(C)** The fraction of values outside (below or above) the PALS suggested cutoff ranges for HR (dark gray) or below the PALS suggested cutoff for sBP (light gray). The dotted line marks the 0.05 line.

We compared cutoff values published in the PALS guidelines (16) and PALS provider manual (17) with our data-derived HR and systolic BP distributions. For HR, we used the ranges cited for awake children as these ranges are wider and were also used by Bonafide et al. (2), while the BP values used were those published in the PALS guidelines as an estimate of the fifth percentile of the systolic BP (2). Figures 4A,B depicts the PALS cutoff values as overlaid (in white lines) on our data-derived distributions for HR and systolic BP. For HR, it can be seen that from 2 years of age onward, a large proportion of the children in the critical care environment exhibit values above what would be considered abnormal by PALS guidelines. On the other hand, the converse is true for BP: as can be seen in Figures 4B,C, the cutoff values suggested by the PALS guidelines actually fall near the fifth percentile for systolic BP for most age groups. Moreover, averaging over the 13 age groups of 0–18 years shows that the difference between the PALS estimated fifth percentile for systolic BP and the one derived in this study is 4.9 ± 3.2 mmHg. Figure 4C continues the comparison to the cutoff values as suggested by the PALS guidelines and shows for each age group the proportion of patients that either lies below the suggested values for systolic BP or outside (i.e., above or below) the range for HR. Indeed, in older children, a large proportion exhibit HRs that would be considered abnormal by the PALS reference values.

**Figure 5:**
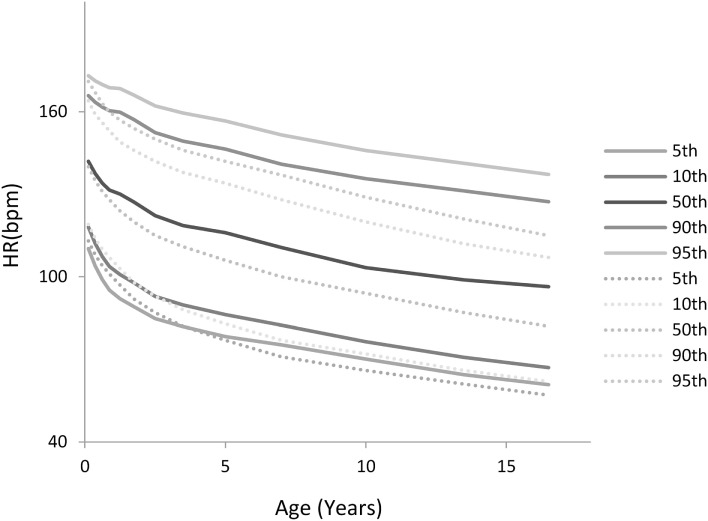
**Comparison of the heart rate (HR) percentiles by age derived in this study with those derived for hospitalized children by Bonafide et al. (2) (dotted lines)**.

Figure 5 shows a comparison of the derived HR percentiles from this study (solid lines) with those recently published for hospitalized children by Bonafide et al. (2) (dotted lines). As can be seen for all age groups, albeit more pronounced for older children, a significant portion in the ICU settings exhibit HRs that are very high relative to what is found in hospitalized children who are not in the PICU.

## Discussion

In this study, we report the development of centile curves for HR and intraarterial BP among children hospitalized in the critical care setting. These distributions were derived from a very large database of physiological variables collected over a 30-month period and comprised hundreds of millions of data points. These centile curves and distributions were compared to previously published reference ranges. We found that a significant portion of the children in the intensive care setting exhibit HRs that are very high relative to PALS cutoff ranges or what is otherwise reported for hospitalized children, whereas the fifth percentile cutoff for systolic BP estimation provided in the PALS guidelines is actually relatively accurate for critically ill children. The percentiles we derived may serve as useful references for pediatric intensivists and serve as a first step toward generating a basis for setting alarm thresholds, clinical trials, and guiding treatment. To the best of our knowledge, this is the first description of distributions of HR and intraarterial BP in children in the critical care settings and mean BP in hospitalized children in general derived from intraarterial BP measurements.

The centile curves reported here were based on all patients admitted to the critical care unit, regardless of the admission or discharge diagnoses, procedures done during the hospitalization, ventilation status, sedation, or inotropic and vasopressor support, similarly to what was previously reported for hospitalized children (2, 3). We fully acknowledge that physiological variables are obviously influenced by potential modifiers as those mentioned above and others such as time from admission or surgery and plan to address their effects in a rigorous prospective manner in future studies. Moreover, it is possible that our finding that systolic BP in this population conforms to the targets suggested by the PALS guidelines reflects the clinicians’ practice to titrate the pharmacologic support to these predefined targets. Due to the offline analytical nature of this study’s design, we could neither test this assumption nor fully characterize the conditional distributions while accounting for all the above-mentioned potential pharmacological and therapeutic modifiers. In that sense, the centile curves reported here are not “normative” data for newborns, infants, and children with critical illness, rather they represent a general estimate to what is the expected HR or BP as a function of age in the critical care settings and serve as a “first pass” in characterizing a diverse population.

Centile curves for any physiological variable essentially plot various percentiles at different ages. Two major statistical challenges involve making these curves: finding different percentiles at each age and achieving smoothness of the estimated percentile curves over age (9). For percentile estimation at each age group, the approach taken by others involves statistical modeling using methods such as generalized linear modeling or generalized additive models for location, scale, and shape (10) to estimate the parameters of a predefined probability distribution thought to best describe the data and from this estimate derive the percentiles. Popular probability distributions chosen to fit physiological and health-related data are the Gaussian (1), Logistic (11), LMS (12), and Box-Cox power exponential (2, 13). In our case, for each age group, millions of measurements were used, several orders of magnitude more than sample sizes used in other similar studies, and we therefore opted to derive the percentiles directly from the data-derived cumulative distributions for each age group. By utilizing millions of observations within each age group, we were able to observe subtle aspects of the distributions that may have otherwise been missed. These subtle features include for example the excess kurtosis in the mean BPs, and the digit preference in the HRs provided by the monitors.

The age groups used for this study were chosen to be consistent with previous studies examining hospitalized children and to allow for maturational changes. Each patient in this study contributed equal weight to an age group distribution, regardless of the total number of corresponding observations, but may have contributed to more than one age group. By using this technique, we could ensure that observations from patients who were monitored for 1 day (accumulating thousands of observations) were given the same weight as patients who stayed in the ICU for several months (accumulating millions of observations).

An important next step in the analysis of the distribution of individual vital signs is to use them to describe the physiologic boundaries, i.e., the physiologic phenotype, for patients admitted to critical care units. It is clearly important to know the target ranges and boundaries for each of the physiologic variables measured, so that clinicians can modify treatment decisions. The target ranges reflect the desired targets that clinicians believe are necessary to demonstrate an appropriate response to treatment, and the boundaries are the upper and lower limits beyond which the clinicians believe is dangerous or critical. Target ranges can be currently set in two ways. One is for the clinical staff to manually set targets based on their preference or preestablished agreements. The other is to use the default ranges for each variable based on “normative data,” data drawn from healthy subjects. The problem with this approach is that the ranges in healthy patients are often not appropriate or normal for patients with critical illness. A fundamental aspect of pediatric critical care medicine is being able to appreciate the evolving clinical picture, and clinicians need to integrate all the available physiologic data being monitored at the bedside with indices of recovery and not blindly focus on keeping a set of physiologic variables within a predetermined range derived from healthy subject.

The strengths of this study lie in the large number of patients and observations used in developing the centile curves. Among its limitations are the facts that all data were collected in a retrospectively single center and that all data points were derived from the automatic bedside monitors and not validated by the bedside nurse. However, automatically obtained data are not affected by human-introduced bias [see, for example, reports on digit preference in reporting respiratory rate by Bonafide et al. (2)], and moreover, cutoff values for alarms, for which the currently reported percentile curves may serve, are also set within the monitors’ automatic data acquisition systems. Another limitation is that we ignored the height of children as this information was not consistently available and because clinicians in the acute setting rarely account for BMI or BSA in their assessment of expected HR or BP. We may be able to assess the impact of these parameters in future work.

The target ranges for specific variables are different based on patient age, size, and end-organ function, and they vary according to diagnosis, procedure, day of recovery, and the clinical management instigated. We did not separate between cardiac and non-cardiac patients nor exclude patients who were receiving vasoactive support. We assumed that clinicians treated the patients to achieve the range and boundaries of HR and BP that they thought was needed during management.

Setting target ranges and boundaries is a dynamic process and should be adjusted regularly and be relevant according to the course of the patient. With the ability to capture and analyze data real time, future work will concentrate on understanding how the ranges and boundaries for vital signs vary in select patient populations to develop an iterative library of patient-specific target ranges and boundaries of physiologic variables.

## Author Contributions

All authors contributed extensively to the work presented in this paper. DE, AG, A-MG, and PL conceived and designed the study. DE, AG, and RG collected and analyzed the data. DE wrote the manuscript, and all authors discussed the results and implications and commented on the manuscript at all stages.

## Conflict of Interest Statement

The authors declare that the research was conducted in the absence of any commercial or financial relationships that could be construed as a potential conflict of interest.
