# Supplementary material for: Heart Rate and Blood Pressure Centile Curves and Distributions by Age of Hospitalized Critically Ill Children
Source: Front Pediatr. 2017 Mar 17;5:52. doi: 10.3389/fped.2017.00052 (PMC5355490; doi:10.3389/fped.2017.00052)
Supplement: Supplementary file 1 [file Table_1.DOCX]

Supplementary Material

**Centile curves and age normative values of heart rate and blood pressure from hospitalized critically ill children**

**Danny Eytan^1,2^, Andrew Goodwin^1^, Anne-Marie Guerguerian^1^, Peter C Laussen^1^**

^1^ Hospital for Sick Children Toronto, Department of Critical Care Medicine, Toronto, Ontario CANADA.

2 Rambam Medical Center, Department of Pediatric Critical Care, Haifa, ISRAEL.

*** Correspondence:** Danny Eytan [d_eytan@rambam.health.gov.il](mailto:d_eytan@rambam.health.gov.il)

Supplementary Material – Table 1 – Heart Rate Percentiles 0-18 years

| **Percentiles**  **Age** | **1** | **5** | **10** | **25** | **50** | **75** | **90** | **95** | **99** |
| --- | --- | --- | --- | --- | --- | --- | --- | --- | --- |
| 0-3 m | 94 | 110 | 118 | 130 | 142 | 155 | 166 | 173 | 188 |
| 3-6 m | 87 | 104 | 112 | 124 | 137 | 151 | 164 | 171 | 187 |
| 6-9 m | 81 | 99 | 107 | 120 | 134 | 148 | 162 | 170 | 185 |
| 9-12 m | 77 | 95 | 104 | 117 | 132 | 146 | 160 | 169 | 184 |
| 12-18 m | 72 | 92 | 101 | 115 | 130 | 145 | 160 | 168 | 184 |
| 18-24 m | 71 | 89 | 98 | 112 | 127 | 143 | 157 | 166 | 183 |
| 2-3 y | 68 | 85 | 93 | 107 | 122 | 138 | 152 | 162 | 182 |
| 3-4 y | 67 | 82 | 90 | 104 | 119 | 134 | 149 | 160 | 179 |
| 4-6 y | 64 | 78 | 86 | 100 | 116 | 131 | 146 | 157 | 174 |
| 6-8 y | 62 | 75 | 82 | 96 | 111 | 126 | 141 | 152 | 170 |
| 8-12 y | 58 | 70 | 76 | 89 | 103 | 120 | 136 | 146 | 165 |
| 12-15 y | 54 | 64 | 71 | 84 | 99 | 115 | 131 | 141 | 160 |
| 15-18 y | 51 | 61 | 67 | 80 | 96 | 111 | 127 | 137 | 155 |
