# Supplementary material for: Heart Rate and Blood Pressure Centile Curves and Distributions by Age of Hospitalized Critically Ill Children
Source: Front Pediatr. 2017 Mar 17;5:52. doi: 10.3389/fped.2017.00052 (PMC5355490; doi:10.3389/fped.2017.00052)
Supplement: Supplementary file 2 [file Table_2.DOCX]

Supplementary Material

**Centile curves and age normative values of heart rate and blood pressure from hospitalized critically ill children**

**Danny Eytan^1,2^, Andrew Goodwin^1^, Anne-Marie Guerguerian^1^, Peter C Laussen^1^**

^1^ Hospital for Sick Children Toronto, Department of Critical Care Medicine, Toronto, Ontario CANADA.

2 Rambam Medical Center, Department of Pediatric Critical Care, Haifa, ISRAEL.

*** Correspondence:** Danny Eytan [d_eytan@rambam.health.gov.il](mailto:d_eytan@rambam.health.gov.il)

Supplementary Material – Table 2 – Heart Rate Percentiles 0-30 Days

| **Percentiles**  **Age (days)** | **1** | **5** | **10** | **25** | **50** | **75** | **90** | **95** | **99** |
| --- | --- | --- | --- | --- | --- | --- | --- | --- | --- |
| 0-1 | 87 | 109 | 116 | 127 | 140 | 151 | 163 | 170 | 184 |
| 1-2 | 87 | 109 | 117 | 129 | 142 | 154 | 165 | 171 | 185 |
| 2-3 | 89 | 110 | 118 | 131 | 144 | 156 | 167 | 173 | 186 |
| 3-4 | 91 | 111 | 120 | 133 | 146 | 158 | 168 | 174 | 187 |
| 4-5 | 93 | 113 | 121 | 134 | 147 | 159 | 169 | 175 | 188 |
| 5-6 | 96 | 114 | 123 | 136 | 149 | 160 | 171 | 176 | 189 |
| 6-7 | 99 | 116 | 125 | 137 | 150 | 162 | 171 | 177 | 190 |
| 7-8 | 104 | 119 | 127 | 139 | 151 | 162 | 172 | 178 | 191 |
| 8-9 | 107 | 121 | 128 | 139 | 151 | 162 | 172 | 178 | 191 |
| 9-10 | 108 | 121 | 128 | 139 | 151 | 162 | 171 | 178 | 191 |
| 10-11 | 107 | 121 | 128 | 139 | 150 | 161 | 171 | 177 | 190 |
| 11-12 | 105 | 121 | 127 | 138 | 150 | 161 | 171 | 177 | 190 |
| 12-13 | 104 | 120 | 127 | 138 | 150 | 161 | 171 | 176 | 190 |
| 13-14 | 103 | 119 | 126 | 137 | 150 | 161 | 171 | 176 | 189 |
| 14-15 | 101 | 119 | 126 | 137 | 150 | 161 | 171 | 176 | 189 |
| 15-16 | 100 | 118 | 125 | 136 | 149 | 161 | 170 | 176 | 188 |
| 16-17 | 99 | 117 | 124 | 136 | 149 | 160 | 169 | 175 | 186 |
| 17-18 | 99 | 117 | 124 | 135 | 148 | 159 | 169 | 174 | 185 |
| 18-19 | 98 | 117 | 124 | 135 | 147 | 159 | 168 | 173 | 184 |
| 19-20 | 98 | 117 | 124 | 135 | 147 | 158 | 168 | 173 | 184 |
| 20-21 | 97 | 117 | 124 | 135 | 146 | 158 | 167 | 173 | 184 |
| 21-22 | 95 | 115 | 123 | 134 | 145 | 157 | 167 | 173 | 184 |
| 22-23 | 93 | 113 | 122 | 133 | 144 | 156 | 166 | 172 | 185 |
| 23-24 | 92 | 112 | 120 | 132 | 144 | 155 | 166 | 172 | 184 |
| 24-25 | 92 | 111 | 120 | 132 | 143 | 155 | 165 | 171 | 184 |
| 25-26 | 93 | 111 | 120 | 132 | 143 | 155 | 165 | 171 | 185 |
| 26-27 | 94 | 111 | 120 | 132 | 143 | 155 | 165 | 171 | 185 |
| 27-28 | 96 | 112 | 120 | 132 | 143 | 155 | 165 | 171 | 185 |
| 28-29 | 98 | 114 | 121 | 132 | 143 | 155 | 165 | 172 | 185 |
| 29-30 | 102 | 117 | 123 | 133 | 144 | 156 | 166 | 173 | 185 |
