# Supplementary material for: Heart Rate and Blood Pressure Centile Curves and Distributions by Age of Hospitalized Critically Ill Children
Source: Front Pediatr. 2017 Mar 17;5:52. doi: 10.3389/fped.2017.00052 (PMC5355490; doi:10.3389/fped.2017.00052)
Supplement: Supplementary file 3 [file Table_3.DOCX]

Supplementary Material

**Centile curves and age normative values of heart rate and blood pressure from hospitalized critically ill children**

**Danny Eytan^1,2^, Andrew Goodwin^1^, Anne-Marie Guerguerian^1^, Peter C Laussen^1^**

^1^ Hospital for Sick Children Toronto, Department of Critical Care Medicine, Toronto, Ontario CANADA.

2 Rambam Medical Center, Department of Pediatric Critical Care, Haifa, ISRAEL.

*** Correspondence:** Danny Eytan [d_eytan@rambam.health.gov.il](mailto:d_eytan@rambam.health.gov.il)

Supplementary Material – Table 3 - Systolic Arterial Blood pressure 0-18 Years

| **Percentiles**  **Age** | **1** | **5** | **10** | **25** | **50** | **75** | **90** | **95** | **99** |
| --- | --- | --- | --- | --- | --- | --- | --- | --- | --- |
| 0-3 m | 45 | 53 | 58 | 66 | 75 | 86 | 98 | 105 | 123 |
| 3-6 m | 52 | 61 | 66 | 74 | 83 | 95 | 108 | 115 | 132 |
| 6-9 m | 57 | 66 | 71 | 79 | 90 | 102 | 115 | 122 | 139 |
| 9-12 m | 60 | 70 | 75 | 83 | 94 | 106 | 119 | 127 | 143 |
| 12-18 m | 61 | 71 | 77 | 84 | 95 | 107 | 120 | 127 | 143 |
| 18-24 m | 60 | 71 | 77 | 85 | 96 | 108 | 120 | 127 | 142 |
| 2-3 y | 58 | 71 | 78 | 88 | 98 | 110 | 122 | 130 | 145 |
| 3-4 y | 63 | 75 | 81 | 89 | 100 | 111 | 123 | 131 | 147 |
| 4-6 y | 67 | 77 | 83 | 91 | 102 | 113 | 124 | 133 | 149 |
| 6-8 y | 66 | 77 | 84 | 94 | 105 | 116 | 128 | 136 | 152 |
| 8-12 y | 65 | 79 | 86 | 97 | 109 | 122 | 136 | 144 | 160 |
| 12-15 y | 65 | 81 | 89 | 100 | 114 | 128 | 143 | 151 | 168 |
| 15-18 y | 68 | 83 | 91 | 103 | 117 | 132 | 146 | 155 | 174 |
