# Supplementary material for: Heart Rate and Blood Pressure Centile Curves and Distributions by Age of Hospitalized Critically Ill Children
Source: Front Pediatr. 2017 Mar 17;5:52. doi: 10.3389/fped.2017.00052 (PMC5355490; doi:10.3389/fped.2017.00052)
Supplement: Supplementary file 4 [file Table_4.DOCX]

Supplementary Material

**Centile curves and age normative values of heart rate and blood pressure from hospitalized critically ill children**

**Danny Eytan^1,2^, Andrew Goodwin^1^, Anne-Marie Guerguerian^1^, Peter C Laussen^1^**

^1^ Hospital for Sick Children Toronto, Department of Critical Care Medicine, Toronto, Ontario CANADA.

2 Rambam Medical Center, Department of Pediatric Critical Care, Haifa, ISRAEL.

*** Correspondence:** Danny Eytan [d_eytan@rambam.health.gov.il](mailto:d_eytan@rambam.health.gov.il)

Supplementary Material – Table 4 - Mean Arterial Blood pressure 0-18 Years

| **Percentiles**  **Age** | **1** | **5** | **10** | **25** | **50** | **75** | **90** | **95** | **99** |
| --- | --- | --- | --- | --- | --- | --- | --- | --- | --- |
| 0-3 m | 35 | 41 | 44 | 49 | 55 | 63 | 71 | 77 | 95 |
| 3-6 m | 39 | 45 | 49 | 54 | 61 | 69 | 80 | 86 | 102 |
| 6-9 m | 42 | 49 | 52 | 58 | 66 | 74 | 86 | 93 | 108 |
| 9-12 m | 43 | 51 | 55 | 61 | 69 | 78 | 90 | 97 | 113 |
| 12-18 m | 45 | 52 | 56 | 62 | 70 | 79 | 91 | 99 | 116 |
| 18-24 m | 44 | 53 | 56 | 62 | 70 | 80 | 90 | 98 | 116 |
| 2-3 y | 45 | 53 | 56 | 63 | 71 | 80 | 90 | 98 | 116 |
| 3-4 y | 47 | 54 | 57 | 63 | 72 | 81 | 90 | 98 | 113 |
| 4-6 y | 48 | 55 | 58 | 65 | 73 | 82 | 92 | 99 | 113 |
| 6-8 y | 48 | 55 | 59 | 66 | 75 | 84 | 94 | 101 | 117 |
| 8-12 y | 49 | 56 | 61 | 68 | 76 | 86 | 95 | 103 | 120 |
| 12-15 y | 49 | 57 | 61 | 69 | 78 | 88 | 97 | 105 | 122 |
| 15-18 y | 50 | 58 | 62 | 69 | 78 | 88 | 98 | 104 | 120 |
