# Supplementary material for: Heart Rate and Blood Pressure Centile Curves and Distributions by Age of Hospitalized Critically Ill Children
Source: Front Pediatr. 2017 Mar 17;5:52. doi: 10.3389/fped.2017.00052 (PMC5355490; doi:10.3389/fped.2017.00052)
Supplement: Supplementary file 5 [file Table_5.DOCX]

Supplementary Material

**Centile curves and age normative values of heart rate and blood pressure from hospitalized critically ill children**

**Danny Eytan^1,2^, Andrew Goodwin^1^, Anne-Marie Guerguerian^1^, Peter C Laussen^1^**

^1^ Hospital for Sick Children Toronto, Department of Critical Care Medicine, Toronto, Ontario CANADA.

2 Rambam Medical Center, Department of Pediatric Critical Care, Haifa, ISRAEL.

*** Correspondence:** Danny Eytan [d_eytan@rambam.health.gov.il](mailto:d_eytan@rambam.health.gov.il)

Supplementary Material – Table 5 - Diastolic Arterial Blood Pressure 0-18 Years

| **Percentiles**  **Age** | **1** | **5** | **10** | **25** | **50** | **75** | **90** | **95** | **99** |
| --- | --- | --- | --- | --- | --- | --- | --- | --- | --- |
| 0-3 m | 27 | 32 | 34 | 38 | 44 | 51 | 59 | 63 | 75 |
| 3-6 m | 28 | 33 | 36 | 40 | 46 | 53 | 62 | 67 | 79 |
| 6-9 m | 30 | 35 | 37 | 42 | 48 | 55 | 64 | 70 | 83 |
| 9-12 m | 31 | 36 | 39 | 43 | 50 | 57 | 67 | 73 | 86 |
| 12-18 m | 32 | 38 | 40 | 45 | 52 | 60 | 70 | 76 | 90 |
| 18-24 m | 32 | 38 | 41 | 46 | 53 | 61 | 70 | 77 | 91 |
| 2-3 y | 33 | 39 | 42 | 47 | 53 | 62 | 71 | 77 | 91 |
| 3-4 y | 34 | 40 | 42 | 48 | 54 | 62 | 72 | 78 | 92 |
| 4-6 y | 35 | 40 | 43 | 49 | 56 | 64 | 73 | 80 | 94 |
| 6-8 y | 36 | 42 | 44 | 50 | 57 | 66 | 75 | 82 | 96 |
| 8-12 y | 37 | 43 | 46 | 52 | 59 | 68 | 77 | 84 | 98 |
| 12-15 y | 37 | 43 | 47 | 53 | 60 | 69 | 78 | 84 | 97 |
| 15-18 y | 37 | 43 | 47 | 53 | 61 | 70 | 79 | 85 | 96 |
