# Supplementary material for: Heart Rate and Blood Pressure Centile Curves and Distributions by Age of Hospitalized Critically Ill Children
Source: Front Pediatr. 2017 Mar 17;5:52. doi: 10.3389/fped.2017.00052 (PMC5355490; doi:10.3389/fped.2017.00052)
Supplement: Supplementary file 6 [file Table_6.DOCX]

Supplementary Material

**Centile curves and age normative values of heart rate and blood pressure from hospitalized critically ill children**

**Danny Eytan^1,2^, Andrew Goodwin^1^, Anne-Marie Guerguerian^1^, Peter C Laussen^1^**

^1^ Hospital for Sick Children Toronto, Department of Critical Care Medicine, Toronto, Ontario CANADA.

2 Rambam Medical Center, Department of Pediatric Critical Care, Haifa, ISRAEL.

*** Correspondence:** Danny Eytan [d_eytan@rambam.health.gov.il](mailto:d_eytan@rambam.health.gov.il)

Supplementary Material – Table 6 - Systolic Arterial Blood pressure 0-30 days

| **Percentiles**  **Age (days)** | **1** | **5** | **10** | **25** | **50** | **75** | **90** | **95** | **99** |
| --- | --- | --- | --- | --- | --- | --- | --- | --- | --- |
| 0-1 | 38 | 42 | 45 | 50 | 57 | 64 | 73 | 79 | 92 |
| 1-2 | 40 | 44 | 47 | 52 | 59 | 67 | 75 | 81 | 94 |
| 2-3 | 41 | 46 | 49 | 54 | 61 | 69 | 77 | 83 | 95 |
| 3-4 | 43 | 48 | 51 | 56 | 63 | 71 | 80 | 85 | 96 |
| 4-5 | 44 | 49 | 52 | 58 | 65 | 73 | 82 | 87 | 98 |
| 5-6 | 44 | 50 | 53 | 60 | 67 | 75 | 83 | 89 | 99 |
| 6-7 | 45 | 51 | 54 | 61 | 68 | 76 | 85 | 90 | 101 |
| 7-8 | 45 | 51 | 55 | 62 | 69 | 77 | 86 | 92 | 102 |
| 8-9 | 46 | 52 | 56 | 63 | 70 | 79 | 87 | 93 | 103 |
| 9-10 | 46 | 53 | 56 | 63 | 71 | 80 | 88 | 94 | 104 |
| 10-11 | 46 | 53 | 57 | 64 | 71 | 80 | 89 | 95 | 105 |
| 11-12 | 45 | 53 | 58 | 64 | 72 | 81 | 90 | 96 | 105 |
| 12-13 | 45 | 54 | 58 | 65 | 73 | 82 | 90 | 96 | 106 |
| 13-14 | 45 | 54 | 59 | 66 | 73 | 82 | 91 | 97 | 108 |
| 14-15 | 46 | 54 | 59 | 66 | 74 | 83 | 91 | 97 | 109 |
| 15-16 | 47 | 55 | 60 | 67 | 75 | 83 | 92 | 98 | 109 |
| 16-17 | 47 | 56 | 61 | 68 | 75 | 84 | 92 | 98 | 110 |
| 17-18 | 48 | 57 | 61 | 69 | 76 | 85 | 93 | 99 | 111 |
| 18-19 | 48 | 57 | 62 | 69 | 76 | 85 | 94 | 100 | 112 |
| 19-20 | 47 | 57 | 62 | 69 | 77 | 86 | 94 | 101 | 113 |
| 20-21 | 46 | 57 | 62 | 69 | 77 | 86 | 95 | 101 | 114 |
| 21-22 | 45 | 56 | 61 | 69 | 77 | 86 | 95 | 101 | 115 |
| 22-23 | 46 | 56 | 61 | 68 | 77 | 86 | 95 | 101 | 114 |
| 23-24 | 48 | 57 | 61 | 69 | 77 | 86 | 94 | 100 | 113 |
| 24-25 | 48 | 57 | 61 | 69 | 77 | 86 | 94 | 100 | 112 |
| 25-26 | 47 | 57 | 61 | 69 | 77 | 86 | 94 | 100 | 111 |
| 26-27 | 45 | 56 | 61 | 69 | 77 | 86 | 94 | 100 | 111 |
| 27-28 | 43 | 56 | 61 | 69 | 77 | 86 | 94 | 100 | 111 |
| 28-29 | 39 | 55 | 61 | 69 | 77 | 86 | 95 | 100 | 112 |
| 29-30 | 35 | 54 | 60 | 68 | 76 | 86 | 95 | 101 | 113 |
