# Supplementary material for: Heart Rate and Blood Pressure Centile Curves and Distributions by Age of Hospitalized Critically Ill Children
Source: Front Pediatr. 2017 Mar 17;5:52. doi: 10.3389/fped.2017.00052 (PMC5355490; doi:10.3389/fped.2017.00052)
Supplement: Supplementary file 7 [file Table_7.DOCX]

Supplementary Material

**Centile curves and age normative values of heart rate and blood pressure from hospitalized critically ill children**

**Danny Eytan^1,2^, Andrew Goodwin^1^, Anne-Marie Guerguerian^1^, Peter C Laussen^1^**

^1^ Hospital for Sick Children Toronto, Department of Critical Care Medicine, Toronto, Ontario CANADA.

2 Rambam Medical Center, Department of Pediatric Critical Care, Haifa, ISRAEL.

*** Correspondence:** Danny Eytan [d_eytan@rambam.health.gov.il](mailto:d_eytan@rambam.health.gov.il)

Supplementary Material – Table 7- Mean Arterial Blood Pressure 0-30 days

| **Percentiles**  **Age (days)** | **1** | **5** | **10** | **25** | **50** | **75** | **90** | **95** | **99** |
| --- | --- | --- | --- | --- | --- | --- | --- | --- | --- |
| 0-1 | 30 | 34 | 36 | 40 | 44 | 50 | 56 | 61 | 73 |
| 1-2 | 31 | 35 | 37 | 41 | 46 | 51 | 57 | 62 | 74 |
| 2-3 | 32 | 37 | 39 | 42 | 47 | 52 | 58 | 63 | 75 |
| 3-4 | 33 | 38 | 40 | 43 | 48 | 53 | 60 | 64 | 75 |
| 4-5 | 34 | 38 | 40 | 44 | 49 | 54 | 61 | 65 | 76 |
| 5-6 | 35 | 39 | 41 | 45 | 49 | 55 | 62 | 66 | 77 |
| 6-7 | 35 | 39 | 41 | 45 | 50 | 56 | 63 | 67 | 78 |
| 7-8 | 35 | 39 | 42 | 45 | 50 | 56 | 63 | 68 | 79 |
| 8-9 | 35 | 39 | 42 | 46 | 51 | 57 | 64 | 69 | 80 |
| 9-10 | 35 | 40 | 42 | 46 | 51 | 57 | 64 | 70 | 81 |
| 10-11 | 35 | 40 | 42 | 46 | 51 | 57 | 64 | 70 | 81 |
| 11-12 | 34 | 40 | 42 | 46 | 51 | 57 | 64 | 70 | 81 |
| 12-13 | 34 | 40 | 43 | 46 | 51 | 57 | 64 | 69 | 81 |
| 13-14 | 34 | 40 | 43 | 47 | 51 | 57 | 64 | 69 | 81 |
| 14-15 | 34 | 40 | 43 | 47 | 51 | 57 | 64 | 69 | 81 |
| 15-16 | 35 | 40 | 43 | 47 | 52 | 57 | 64 | 69 | 81 |
| 16-17 | 35 | 41 | 43 | 47 | 52 | 58 | 65 | 70 | 83 |
| 17-18 | 36 | 41 | 44 | 47 | 52 | 58 | 65 | 71 | 84 |
| 18-19 | 37 | 41 | 43 | 47 | 53 | 59 | 66 | 71 | 85 |
| 19-20 | 37 | 41 | 43 | 47 | 53 | 59 | 66 | 72 | 86 |
| 20-21 | 36 | 41 | 43 | 47 | 53 | 59 | 66 | 71 | 86 |
| 21-22 | 35 | 40 | 42 | 47 | 53 | 59 | 66 | 71 | 85 |
| 22-23 | 34 | 40 | 42 | 47 | 52 | 58 | 66 | 71 | 85 |
| 23-24 | 34 | 40 | 43 | 47 | 52 | 59 | 66 | 71 | 84 |
| 24-25 | 34 | 40 | 43 | 47 | 52 | 59 | 67 | 72 | 84 |
| 25-26 | 34 | 40 | 43 | 47 | 53 | 59 | 67 | 72 | 84 |
| 26-27 | 33 | 40 | 43 | 47 | 53 | 59 | 68 | 73 | 85 |
| 27-28 | 32 | 40 | 42 | 47 | 53 | 60 | 68 | 73 | 85 |
| 28-29 | 31 | 40 | 42 | 47 | 53 | 60 | 69 | 74 | 86 |
| 29-30 | 31 | 39 | 42 | 47 | 53 | 61 | 69 | 75 | 87 |
