# Supplementary material for: Heart Rate and Blood Pressure Centile Curves and Distributions by Age of Hospitalized Critically Ill Children
Source: Front Pediatr. 2017 Mar 17;5:52. doi: 10.3389/fped.2017.00052 (PMC5355490; doi:10.3389/fped.2017.00052)
Supplement: Supplementary file 8 [file Table_8.DOCX]

Supplementary Material

**Centile curves and age normative values of heart rate and blood pressure from hospitalized critically ill children**

**Danny Eytan^1,2^, Andrew Goodwin^1^, Anne-Marie Guerguerian^1^, Peter C Laussen^1^**

^1^ Hospital for Sick Children Toronto, Department of Critical Care Medicine, Toronto, Ontario CANADA.

2 Rambam Medical Center, Department of Pediatric Critical Care, Haifa, ISRAEL.

*** Correspondence:** Danny Eytan [d_eytan@rambam.health.gov.il](mailto:d_eytan@rambam.health.gov.il)

Supplementary Material – Table 8 - Diastolic Arterial Blood Pressure 0-30 days

| **Percentiles**  **Age (days)** | **1** | **5** | **10** | **25** | **50** | **75** | **90** | **95** | **99** |
| --- | --- | --- | --- | --- | --- | --- | --- | --- | --- |
| 0-1 | 22 | 26 | 28 | 32 | 36 | 41 | 46 | 51 | 57 |
| 1-2 | 23 | 27 | 29 | 32 | 36 | 42 | 47 | 51 | 59 |
| 2-3 | 23 | 27 | 29 | 33 | 37 | 42 | 48 | 52 | 60 |
| 3-4 | 24 | 28 | 30 | 33 | 38 | 43 | 49 | 53 | 61 |
| 4-5 | 24 | 28 | 30 | 33 | 38 | 44 | 49 | 53 | 62 |
| 5-6 | 24 | 28 | 31 | 34 | 39 | 44 | 50 | 54 | 62 |
| 6-7 | 25 | 29 | 31 | 34 | 39 | 45 | 50 | 54 | 63 |
| 7-8 | 25 | 29 | 31 | 34 | 39 | 45 | 51 | 55 | 64 |
| 8-9 | 25 | 29 | 31 | 34 | 39 | 45 | 51 | 56 | 64 |
| 9-10 | 25 | 29 | 31 | 34 | 39 | 45 | 51 | 56 | 65 |
| 10-11 | 26 | 29 | 31 | 34 | 39 | 45 | 51 | 56 | 65 |
| 11-12 | 26 | 29 | 30 | 34 | 39 | 44 | 51 | 55 | 64 |
| 12-13 | 25 | 29 | 30 | 34 | 38 | 44 | 50 | 55 | 64 |
| 13-14 | 25 | 28 | 30 | 33 | 38 | 44 | 50 | 55 | 64 |
| 14-15 | 25 | 28 | 30 | 33 | 38 | 44 | 50 | 54 | 63 |
| 15-16 | 24 | 27 | 29 | 33 | 38 | 44 | 50 | 54 | 63 |
| 16-17 | 24 | 27 | 29 | 33 | 38 | 44 | 50 | 55 | 64 |
| 17-18 | 24 | 27 | 29 | 33 | 38 | 45 | 51 | 56 | 65 |
| 18-19 | 24 | 27 | 29 | 33 | 38 | 45 | 51 | 56 | 66 |
| 19-20 | 24 | 27 | 29 | 33 | 38 | 45 | 51 | 56 | 66 |
| 20-21 | 24 | 27 | 29 | 32 | 38 | 44 | 51 | 56 | 66 |
| 21-22 | 23 | 27 | 28 | 32 | 38 | 44 | 51 | 56 | 66 |
| 22-23 | 23 | 26 | 28 | 32 | 38 | 44 | 51 | 56 | 66 |
| 23-24 | 22 | 26 | 28 | 32 | 38 | 44 | 51 | 56 | 67 |
| 24-25 | 22 | 26 | 28 | 32 | 38 | 44 | 51 | 57 | 67 |
| 25-26 | 22 | 26 | 28 | 32 | 38 | 45 | 52 | 57 | 67 |
| 26-27 | 22 | 26 | 28 | 32 | 38 | 45 | 52 | 58 | 67 |
| 27-28 | 22 | 26 | 28 | 32 | 39 | 46 | 53 | 58 | 68 |
| 28-29 | 23 | 26 | 28 | 32 | 39 | 47 | 53 | 59 | 68 |
| 29-30 | 23 | 26 | 28 | 32 | 39 | 47 | 54 | 59 | 69 |
